# Dickkopf-3 in the prediction of contrast media induced acute kidney injury

**DOI:** 10.1007/s40620-020-00910-1

**Published:** 2020-12-04

**Authors:** Felix S. Seibert, Anja Heringhaus, Nikolaos Pagonas, Benjamin Rohn, Frederic Bauer, Hans-Joachim Trappe, Ulf Landmesser, Nina Babel, Timm H. Westhoff

**Affiliations:** 1 Department of Nephrology, University Hospital Marien Hospital Herne, Ruhr-University of Bochum, Hölkeskampring 40, 44625 Herne, Germany; 2 Department for Angiology, Centre for Internal Medicine I, Brandenburg Medical School Theodor Fontane, Brandenburg, Germany; 3 Department of Cardiology, University Hospital Marien Hospital Herne, Ruhr-University of Bochum, Herne, Germany; 4 Department of Cardiology, Charité – Campus Benjamin Franklin, Berlin, Germany

**Keywords:** Dickkopf-3, Contrast media induced acute kidney injury, CI-AKI, Coronary angiography, Tubular toxicity

## Abstract

**Background:**

Dickkopf-3 (DKK3) has recently been discovered as a urinary biomarker for the prediction of acute kidney injury (AKI) after cardiac surgery. This finding needs to be confirmed for AKI in other clinical settings. The present study investigates whether DKK3 can predict contrast-induced AKI (CI-AKI).

**Methods:**

We performed a prospective study in 490 patients undergoing coronary angiography. Primary endpoint was an increase in serum creatinine concentration ≥ 0.3 mg/dl within 72 h after the procedure. DKK3 was assessed < 24 h before coronary angiography. Predictive accuracy was assessed by receiver operating characteristic (ROC) curves.

**Results:**

CI-AKI was observed in 30 (6.1%) patients, of whom 27 corresponded to stage I and 3 to stage II according to the Acute Kidney Injury Network (AKIN) criteria. Subjects who developed CI-AKI had a 3.8-fold higher urinary DKK3/creatinine ratio than those without CI-AKI (7.5 pg/mg [interquartile range [IQR] 1.2–1392.0] vs. 2.0 pg/mg [IQR 0.9–174.0]; p = 0.047). ROC analysis revealed an area under the curve (AUC) of 0.61. Among subjects without clinically overt chronic kidney disease (estimated glomerular filtration rate [eGFR] > 60 ml/min, urinary albumin creatinine ratio < 30 mg/g), the DKK3/creatinine ratio was 5.4-fold higher in those with subsequent CI-AKI (7.5 pg/mg [IQR 0.9–590.1] vs. 1.38 pg/mg [IQR 0.8–51.0]; *p* = 0.007; AUC 0.62). Coronary angiography was associated with a 43 times increase in the urinary DKK3/creatinine ratio.

**Conclusions:**

Urinary DKK3 is an independent predictor of CI-AKI even in the absence of overt chronic kidney disease (CKD). The study thereby expands the findings on DKK3 in the prediction of postoperative loss of kidney function to other entities of AKI.

**Graphic abstract:**

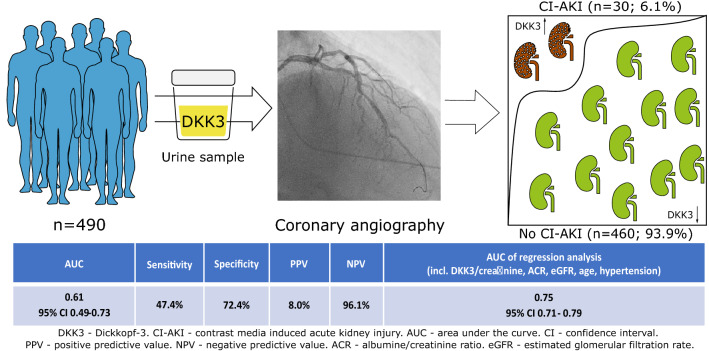

## Introduction

Dickkopf-3 (DKK3) is a recently discovered stress-induced tubular-derived renal biomarker for interstitial fibrosis [1]. Thus, it was speculated that increased urinary DKK3 concentrations could indicate a higher risk of progression of chronic kidney disease (CKD). This association has indeed been demonstrated in the participants of the STOP-IgAN trial [2]. Beyond CKD, there is a first investigation on DKK3 in the context of acute kidney injury (AKI). In subjects undergoing cardiac surgery preoperative urinary DKK3 concentrations had a prognostic value for the development of postoperative AKI [3]. Thus, DKK3 may be able to identify subjects at increased risk who could benefit from preventive measures. The findings of this prominently published study raise the question of whether this could also apply to other clinical settings associated with the risk of AKI of other origins. Moreover, the sensitivity of DKK3 in the prediction of AKI has not been defined. Cardiac surgery may be associated with severe tubular damage. Is DKK3 also able to identify subjects who will develop milder forms of AKI? Although the controversial debate on the impact of contrast media on kidney function is still ongoing, it is generally accepted that the effect on kidney function is rather mild in the majority of cases. Thus, contrast induced AKI (CI-AKI) may be a suitable model for investigating whether DKK3 is able to predict mild deterioration of glomerular filtration rate (GFR) as well. The present prospective study therefore investigates the association of pre-procedural urinary DKK3 concentrations with subsequent CI-AKI in subjects undergoing coronary angiography. It is thereby the first validation study of the above mentioned proof of principle study and examines the potential prediction of AKI by DKK3 in the setting of cardiac catheterization, one of the most frequently performed clinical procedures.

## Materials and methods

### Study design and protocol

The present work constitutes a prospective bi-center study performed at the University Hospital Marien Hospital Herne (Ruhr-University of Bochum) and Charité University Medicine Berlin—Campus Benjamin Franklin. The study was approved by the local ethics committee of the Ruhr-University Bochum (registry number 4866–13) and the Charité—Universitätsmedizin Berlin (registry number EA4/117/13). All patients provided written informed consent.

Patients with elective indication for a coronary angiography (CA) were enrolled in the study. Exclusion criteria were hypotension (e.g. cardiogenic shock), obstructive uropathy, urothelial carcinoma, metastatic cancer, and pyuria with semiquantitative dipstick examination > 1. Pre-existing CKD was defined according to KDIGO criteria [4]. Subjects with an estimated GFR (eGFR) < 60 ml/min and/or albuminuria > 30 mg/g creatinine were categorized as "clinically overt kidney disease". Physicians were blinded to the results of biomarker assessments. The volume of contrast media was documented. Preventive plasma expansion was performed according to the physicians’ clinical assessment with 1000 ml of normochloraemic solution (Jonosteril, Fresenius, Germany) right before (< 2 h) and after (< 2 h) coronary angiography. Blood and urine samples were collected 24 h before coronary angiography and eventual plasma expansion. A second sample of urine and blood was obtained 48 to 72 h after contrast media. Both urine samples were analyzed for DKK3, albumin and creatinine concentrations. eGFR was calculated by means of the MDRD formula and AKI was defined according to Acute Kidney Injury Network (AKIN) criteria [5]. Data about other urinary biomarkers in this population have been published separately, including a detailed description of the baseline parameters of the study population [6].

### Measurement of urinary DKK3, albumin and creatinine concentrations

Urine samples (10 ml) were collected and stored frozen (-20 °C) until measurement of biomarker concentrations. Urinary concentration of DKK3 (ReFiNE; DiaRen UG, Homburg/Saar, Germany) was assessed by enzyme-linked immunosorbent assay (ELISA) according to the manufacturer’s protocol and previous publications [2, 7, 8]. Albumin was assessed turbidimetrically (ALBU2, Roche Diagnostics), and creatinine was obtained by enzymatic assay (CRE2U, Roche Diagnostics). DKK3 and albumin concentrations were normalized to urinary creatinine concentrations.

### Statistical analysis

Data were analyzed for Gaussian distribution (D’Agostino Pearson) and are presented as median and interquartile range (IQR). Intergroup differences of numerical data were tested for statistical significance non-parametrically by the Mann Whitney U test. Receiver-operating characteristic (ROC) curves were formed in an attempt to determine the accuracy of the urinary DKK3/creatinine ratio in predicting CI-AKI (area under the curve [AUC]). Youden’s index (*J* = sensitivity + specificity—1) was used to establish the best threshold in this differentiation. We performed logistic regression testing for CI-AKI including the predictors DKK3/creatinine ratio, ACR (except for the non CKD cohort), eGFR and age and hypertension. The concordance statistic result of the model is expressed as AUC. After an initial statistical analysis of the overall study population, procedures were repeated for the population without pre-existing clinically overt CKD and those without preventive plasma expansion. *p* < 0.05 was considered statistically significant. All statistical analyses were performed using Prism 7 (GraphPad Software, La Jolla, CA, USA), SPSS Statistics 25 (SPSS Inc., Chicago, IL, USA) and MedCalc (MedCalc Software Ltd., Ostend, Belgium).

## Results

Four hundred and ninety patients were enrolled and received a follow-up examination 48–72 h after coronary angiography. The study population comprised 363 male and 127 female patients with a median age of 66 (IQR 57–73). Median eGFR was 78.6 (IQR 57–73). The main comorbidities were hypertension (78.8%), coronary heart disease (65.1%) and diabetes (25.7%). Three hundred and forty-six patients did not suffer from pre-existing CKD, comprising 266 male and 80 female patients with a median age of 61 (IQR 54–70). Peri-procedural plasma expansion was judged unnecessary in 370 subjects (75.5%). This subgroup included 278 males and 92 females with a median age of 63 (IQR 55–71). Assessment of urinary DKK3 was unsuccessful in 3 samples. Data concerning follow-up urine after coronary angiography were missing in 14 cases. Further population characteristics are presented in Table 1.

**Table 1:** Population characteristics of CI-AKI and no CI-AKI

	CI-AKI	NO CI-AKI	*p*
Total study population; *n* = 490	30 (6.1%)	460 (93.9%)	
Age	71 (63–76)	65 (56–73)	**0.013**
Body mass index (kg/m^2^)	28.7 (24.8–31.8)	28.4 (25.7–32.4)	0.664
Female	9 (30.0%)	118 (25.6%)	0.667
Male	21 (70.0%)	342 (74.4%)	
eGFR (ml/min)	69.7 (52.9–92.0)	78.8. (63.6–91.8)	0.197
ACR (mg/g creatinine)	19.8 (8.2–57.3)	5.2 (2.9–15.1)	**0.001**
Hypertension	30 (100.0%)	356 (77.4%)	**0.001**
Diabetes	10 (33.3%)	116 (25.2%)	0.386
Smoking	9 (30.0%)	214 (46.5%)	0.089
Hyperlipidemia	17 (56.7%)	217 (47.2%)	0.349
Uremic acid (mg/dl)	6.6 (5.4–7.5)	6.7 (5.1–7.1)	0.208
Contrast media (ml)	80 (60–125)	80 (60–120)	0.959
Subgroup without CKD; *n* = 346	15 (4.3%)	331 (95.7%)	
Age	70 (59–72)	61 (54–69)	0.130
BMI	26.8 (25.1–31.8)	28.4 (25.7–32.3)	0.562
Female	3 (20.0%)	77 (23.3%)	0.999
Male	12 (80.0%)	254 (76.7%)	
eGFR (ml/min)	83.4 (66.3–105.2)	81.9 (73.9–94.4)	0.976
ACR (mg/g creatinine)	13.9 (3.5–20.1)	4.2 (2.7–7.5)	0.060
Hypertension	15 (100.0%)	242(73.1%)	**0.015**
Diabetes	2 (13.3%)	66 (19.9%)	0.744
Smoking	6 (40.0%)	165 (49.9%)	0.599
Hyperlipidemia	9 (60.0%)	161 (48.6%)	0.438
Uremic acid (mg/dl)	6.2 (4.8–7.2)	5.9 (5.0–6.9)	0.949
Contrast media (ml)	70 (60–120)	80 (60–120)	0.649
Subgroup without plasma expansion; *n* = 370	17 (4.6%)	353 (95.4%)	
Age	70 (62–74)	63 (55–71)	0.091
BMI (kg/m^2^)	26.8 (24.2–32.8)	28.7 (25.8–33.0)	0.444
Female	4 (23.5%)	88 (24.9%)	0.999
Male	13 (76.5%)	265 (75.1%)	
eGFR (ml/min)	83.4 (66.2–103.4)	80.7 (70.5–93.8)	0.863
ACR (mg/g creatinine)	13.6 (3.6–19.2)	4.7 (2.8–10.5)	0.133
Hypertension	17 (100.0%)	263 (74.5%)	**0.017**
Diabetes	5 (29.4%)	76 (21.5%)	0.546
Smoking	7 (41.2%)	172 (48.7%)	0624
Hyperlipidemia	11 (64.7%)	167 (47.3%)	0.215
Uremic acid (mg/dl)	6.2 (5.1–7.1)	5.9 (5.0–7.0)	0.634
Contrast media (ml)	60 (60–110)	80 (60–113)	0.516

*CI-AKI* contrast media induced acute kidney injury, *eGFR* estimated glomerular filtration rate, *ACR* albumin/creatinine ratio, *CKD* chronic kidney disease

**Fig. 1:**
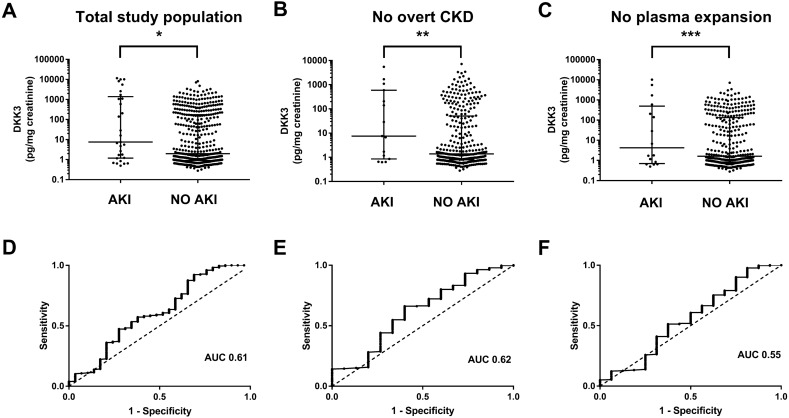
Individual urinary DKK3/creatinine ratios of subjects with and without contrast media-induced acute kidney injury (CI-AKI) after coronary angiography and the corresponding ROC analysis in **a**, **d** the overall study population, **b**, **e** the subgroup population without overt chronic kidney disease (CKD), and **c**, **f** without plasma expansion prior to coronary angiography. Data are presented as scatter plots (logarithmic Y-axis; medians and interquartile ranges are indicated by horizontal lines). Significant differences were ***p < 0.001, **p < 0.01 and *p < 0.05 by Mann–Whitney testing. Diagonal scattered lines indicate prediction of CI-AKI by chance. *AUC* area under the curve

CI-AKI occurred in 30 patients (6.1%). Three patients (0.6%) were classified as AKIN stage II and 27 (5,5%) as stage I. None of the patients suffered from AKIN stage III. Patients with CI-AKI had a 3.8-fold higher pre-procedural DKK3/creatinine ratio than those without AKI after intravenous contrast media application (7.5 pg/mg [IQR 1.2–1392.0] vs. 2.0 pg/mg [IQR 0.9–174.0]; *p* = 0.047). The accuracy of urinary DKK3/creatinine in the detection of CI-AKI was assessed by ROC curve analysis, yielding an AUC of 0.61 (95% CI 0.49–0.73). Data are presented in Fig. 1. Table 2 presents the predictive accuracy using the optimal cut-off values obtained by Youden index. The best cut-off value for DKK3 was 1.7 pg/mg creatinine (*J* = 0.198), computing 47.4% sensitivity, 72.4% specificity, 8.0% positive predictive value (PPV) and 96.1% negative predictive value (NPV). Albumin/creatinine ratio was 4 times higher in patients, who suffered from CI-AKI (19.8 mg/g [IQR 8.2–57.3] vs. 5.2 mg/g [IQR 2.9–15.1]; *p* = 0.001). The product of ACR with DKK3 led to an AUC of 0.65 (95% CI 0.55–0.76). Calculation for DKK3•ACR showed 57.2% sensitivity, 72.4% specificity, 9.7% PPV and 96.7% NPV. The logistic regression model for the development of CI-AKI, using the parameters of DKK3/creatinine, albumin/creatinine, eGFR and age and hypertension, provided an AUC of 0.75 (95% CI 0.71–0.79).

**Fig. 2:**
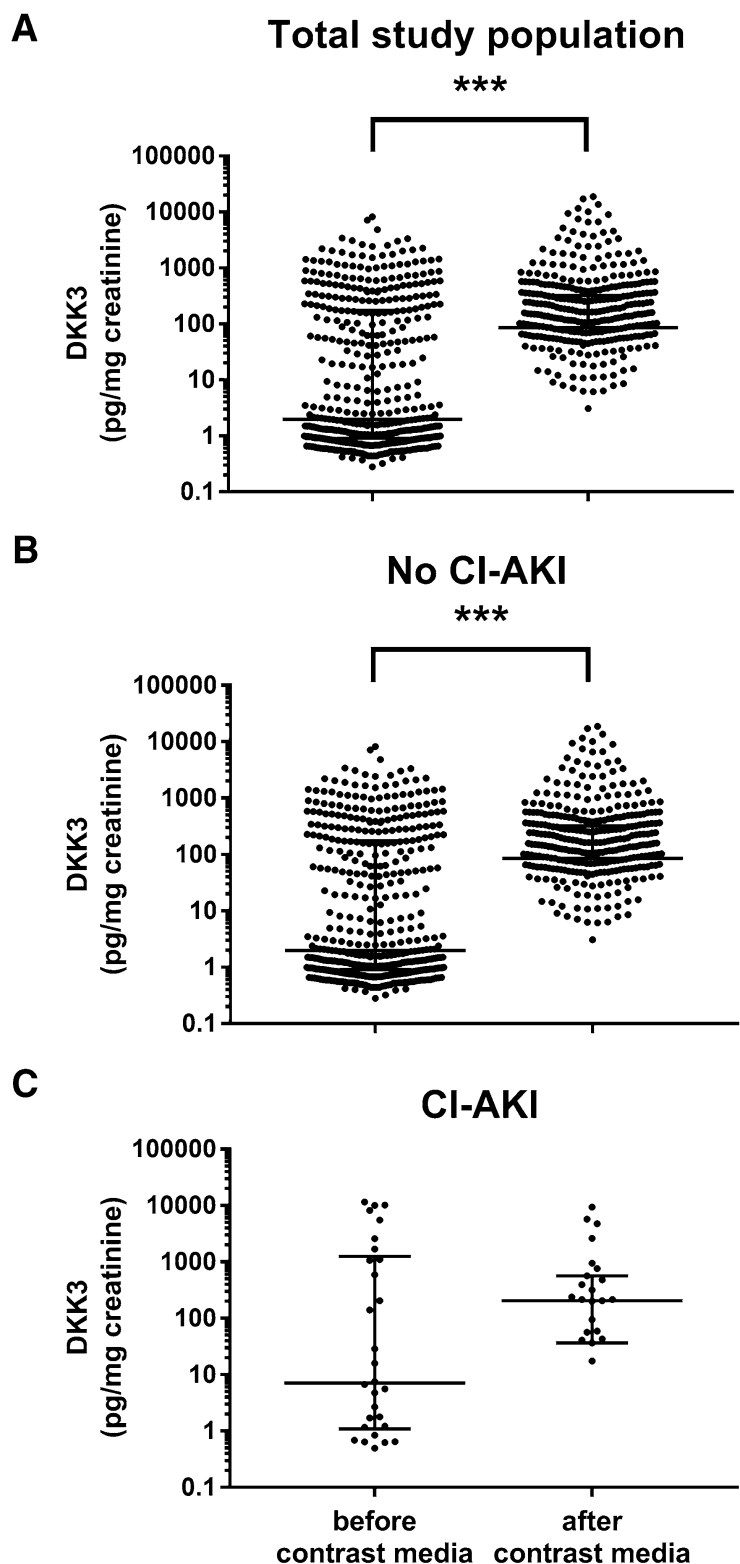
Development of individual urinary DKK3/creatinine ratios before and after coronary angiography in **a** the overall study population, **b** the subgroup of patients without CI-AKI and **c** those who suffered from CI-AKI. Data are presented as scatter plots (logarithmic Y-axis; medians and interquartile ranges are indicated by horizontal lines). Significant differences were ***p < 0.001, **p < 0.01 and *p < 0.05 by Wilcoxon paired testing. CI-AKI – contrast media-induced acute kidney injury

**Table 2:** Diagnostic performance of urinary DKK3/creatinine ratios prior to coronary angiography

	Total study population *n* = 490	Subgroup without CKD *n* = 346	Subgroup without plasma expansion *n* = 370
Urinary DKK3/creatinine CI-AKI (pg/mg crea)	7.5 (1.2–1392.0)	7.5 (0.9–590.1)	4.3 (0.7–494.2)
Urinary DKK3/creatinine NO CI-AKI (pg/mg crea)	2.0 (0.9–174.0)	1.4 (0.8–51.0)	1.6 (0.8–132.2)
*p*	**0.047**	**0.007**	**0.001**
AUC 95% CI	0.61 0.49–0.73	0.62 0.46–0.78	0.55 0.39–0.72
Sensitivity	47.4%	66.2%	51.2%
Specificity	72.4%	60.0%	62.5%
PPV	8.0%	7.4%	5.5%
NPV	96.1%	97.3%	96.3%
AUC of regression analysis (incl. DKK3/creatinine, ACR, eGFR, age, hypertension)	0.75 95% CI 0.71–0.79	0.77 95% CI 0.72–0.81	0.74 95% CI 0.69–0.78

Interquartile range of numeric data are presented in brackets

*AUC* area under the curve, *CI* confidence interval, *DKK3* Dickkopf-3, *eGFR* estimated glomerular filtration rate, *ACR* albumin/creatinine ratio, *PPV* positive predictive value, *NPV* negative predictive value

In a second approach, the low risk cohort of patients without pre-existing clinically overt CKD (*n* = 346) was investigated. Median eGFR was 81.9 ml/min (IQR 73.7–94.5) with an ACR of 4.2 mg/g creatinine (IQR 2.7–7.8). Contrast media volume was comparable to the overall population (80 ml [IQR 60–120] vs. 80 ml [60–120]; p = 0.6). Amongst these 346 patients, 15 developed CI-AKI (4.3%), the severity of which was mild (14 AKIN I [4.0%], 1 case of AKIN II [0.3%]). As described in Table 2, urinary DKK3/creatinine ratio was significantly higher in subjects with subsequent CI-AKI (7.5 pg/mg [IQR 0.9–590.1] vs. 1.4 pg/mg [IQR 0.8–51.0]; *p* = 0.007). ROC analysis computed an AUC of 0.62 (95% CI 0.46–0.78). The best cut-off by Youden index for DKK3 was 6.6 pg/mg creatinine (*J* = 0.262), computing 66.2% sensitivity, 60.0% specificity, 7.4% PPV and 97.3% NPV. Regression analysis implementing DKK3/creatinine, eGFR, age and hypertension showed an AUC of 0.77 (95% CI 0.72–0.81).

In subjects who did not receive preventive fluid administration (*n* = 370), CI-AKI occurred 17 (4.6%) times, including 1 (0.3%) that was classified as AKIN II and 16 as AKIN I (4.3%). The DKK3/creatinine ratio showed significantly higher concentrations in individuals with CI-AKI (4.3 pg/mg [IQR 0.7–494.2]) compared to those without CI-AKI (1.6 pg/mg [IQR 0.8–132.2]; p = 0.001), yielding an AUC of 0.55 (95% CI 0.39–0.72; Table 2). Using cut-off values obtained by Youden index (*J* = 0.138; cut-off 1.7 pg/mg creatinine), sensitivity, specificity, PPV and NPV were 51.2%, 62.5%, 5.5% and 96.3%, respectively. ROC analysis of DKK3•ACR in this subgroup led to an AUC of 0.58 (95% CI 0.44–0.73). PPV and NPV were 6.7% and 97.8%, respectively. Logistic regression including DKK3/creatinine, albumin/creatinine, eGFR, age and hypertension computed an AUC of 0.74 (95% CI 0.69–0.78). Furthermore, the remaining 120 patients, who were subject to normochloraemic crystalloid infusion, showed a significantly higher urinary DKK3/creatinine ratio of 6.2 (IQR 1.4–573.1) pg/mg creatinine compared to those who were less likely to develop an adverse renal event (1.6 [IQR 0.8–147.3]) pg/mg creatinine, *p* < 0.001).

We analyzed the change in DKK3/creatinine ratios in the overall population from baseline to follow-up. DKK3 ratios were 42-fold higher after coronary angiography (2.1 pg/mg [IQR 0.9–187.9] vs. 89.5 pg/mg [IQR 0–338.3]), *p* = 0.001). The rise of DKK3/creatinine in subjects without apparent renal impairment (no CI-AKI according to AKIN criteria) was 43-fold (2.0 pg/mg [IQR 0.9–174] vs. 86.1 pg/mg [IQR 0–322.6]; *p* = 0.001). Those with CI-AKI (7.1 pg/mg [IQR 1.1–1245] vs. 206.1 pg/mg [IQR 36.6–567.4]; *p* = 0.58) presented a 29-fold increase in DKK3/creatinine ratios (Fig. 2).

## Discussion

The novel renal biomarker DKK3 has previously been demonstrated to be an independent predictor of AKI after cardiac surgery [3]. The present study investigated for the first time the prognostic value of DKK3 in the context of coronary angiography as a setting with expected lower rates and milder forms of AKI. DKK3 indeed proved to be a predictor of AKI after coronary angiography. The prognostic accuracy, however, was somewhat lower than for post-operative AKI.

The rate of AKI was 6.1% in the present study. The National Cardiovascular Data Registry (NCDR) Cath-PCI registry revealed an overall CI-AKI incidence of 7.1%, with 0.3% requiring dialysis [9]. Other studies of the NCDR Cath-PCI registry since have demonstrated similar rates of CI-AKI between 7 and 8% [10, 11]. Thus, the present AKI rate was lower as expected. Nevertheless, the study was sufficiently powered to detect a significant 3.8 times difference in subjects who developed post-procedural AKI. It has to be kept in mind that AKI after coronary angiography may not solely be mediated by contrast media. Cholesterol embolisms may subordinately contribute to a deterioration of renal function.

Interestingly, DKK3 concentrations are significantly higher in subjects without clinically overt CKD and who are also prone to CI-AKI. Thus, DKK3 may help to identify subjects at increased risk who would generally not receive peri-procedural preventive measures like plasma expansion to avoid CI-AKI. Although this is a promising result, the prognostic accuracy with an AUC of 0.62 is too low to suggest an introduction into clinical practice at present. Compared to a of sensitivity 76.0% and specificity of 79.1% for predicting postoperative AKI, the accuracy in the present prediction of CI-AKI revealed a Youden index-based sensitivity of only 47.4% and a specificity of 72.4% [12]. Population characteristics revealed the outstanding fact that every patient with CI-AKI suffered from pre-existing hypertension, which contrasts with patients who did not meet renal impairment after contrast media application. We therefore included hypertension into our regression analysis with standard variables, which led to a moderate AUC of 0.75. But why is the prognostic accuracy worse than for AKI after cardiac surgery? The most likely explanation is the mild expression of AKI with 5.3% of the subjects suffering from AKI stage I and none with stage III in the overall study population. The less injurious an intervention, the lower the discriminatory potential of a biomarker. Tubular damage in the context of cardiac surgery exceeds the mean damage by the administration of contrast media. Nevertheless, DKK3 has proven to be an independent predictor even in this setting.

The present work constitutes the first prospective study investigating the predictive value of DKK3 in AKI beyond the context of cardiac surgery. Hence, there are two major conclusions: First, the prognostic information of DKK3 is not limited to one single kind of AKI. Second, the provide a first necessary confirmation of the proof of principle study. The findings thereby expand our knowledge on this promising new biomarker. Future interventional studies are necessary to investigate whether this prognostic information can be translated into a reduction of the incidence of AKI.

The low predictive value of GFR in the present study is surprising at first sight. This finding should likely be attributed to preventive plasma expansion. The decision for or against plasma expansion is mainly driven by serum creatinine. It may be assumed that the administration of fluid led to a transient increase in GFR with a subsequently lower rate of AKI. This issue is a major limitation of the present trial. However, we decided against a restriction of plasma expansion both for ethical reasons and in order to investigate potential additive information by DKK3 in a real life scenario. Interestingly, the simultaneous use of albumin and DKK3—as done by the product of albumin/creatinine and DKK3/creatinine—was able to augment the discriminatory potential, albeit just slightly. A further limitation is the fact that GFR was calculated by MDRD formula, which is standard of care in our institution.

DKK3 has pro-fibrotic properties, promoting renal tubulointerstitial fibrosis through modulation of the canonical Wnt/b-catenin signaling pathway [1]. In clinical studies, increased urinary DKK3 levels identified patients at high risk for short-term CKD progression, regardless of the cause of kidney disease, baseline kidney function and albuminuria. Our data showed increased DKK3/creatinine concentrations after application of contrast media, independently of the occurrence of CI-AKI. With regard to the pathogenic potential of DKK3, we speculate that a high urinary DKK3 ratio mirrors subclinical renal injury. Whether this acute increase in DKK3 indicates an increased risk of long-term deterioration of kidney function is beyond the scope of the present study.

Beyond the administration of contrast media or cardiac surgery, there are other clinical scenarios that inhibit an increased risk of AKI, e.g. intensive care medicine. The present findings show that DKK3 may serve as a helpful adjunct to identify subjects with increased tubular vulnerability, even though there is no albuminuria or impaired GFR. In 2005 the American Society of Nephrology called for the identification and characterization of renal biomarkers in order to improve the prevention and management of AKI. Since that time several biomarkers like neutrophil gelatinase-associated lipocalin (NGAL), kidney injury molecule-1 (KIM-1) or tissue inhibitor of metalloproteinases 2 • insulin-like growth factor-binding protein 7 (TIMP-2•IGFBP7) have been extensively investigated. Despite many interesting and promising findings their introduction into daily clinical practice is low. The future will show whether the prognostic potency of DKK3 in both CKD and AKI will allow a more favorable evolution.

In conclusion, the present study shows that DKK3 is an independent predictor of AKI after coronary angiography. It thereby confirms the findings of the pilot study and illustrates that it expands our knowledge on DKK3 in an alternative kind of AKI. The prognostic accuracy is lower, however, than in the setting of cardiac surgery, which does not allow us to recommend its implementation into clinical practice for the prevention of CI-AKI at present.

## Data Availability

The dataset generated during the current study is available on a data repository.
